# A Practical Comparison of *De Novo* Genome Assembly Software Tools for Next-Generation Sequencing Technologies

**DOI:** 10.1371/journal.pone.0017915

**Published:** 2011-03-14

**Authors:** Wenyu Zhang, Jiajia Chen, Yang Yang, Yifei Tang, Jing Shang, Bairong Shen

**Affiliations:** Center for Systems Biology, Soochow University, Suzhou, Jiangsu, China; Georgia Institute of Technology, United States of America

## Abstract

The advent of next-generation sequencing technologies is accompanied with the development of many whole-genome sequence assembly methods and software, especially for *de novo* fragment assembly. Due to the poor knowledge about the applicability and performance of these software tools, choosing a befitting assembler becomes a tough task. Here, we provide the information of adaptivity for each program, then above all, compare the performance of eight distinct tools against eight groups of simulated datasets from Solexa sequencing platform. Considering the computational time, maximum random access memory (RAM) occupancy, assembly accuracy and integrity, our study indicate that string-based assemblers, overlap-layout-consensus (OLC) assemblers are well-suited for very short reads and longer reads of small genomes respectively. For large datasets of more than hundred millions of short reads, *De Bruijn* graph-based assemblers would be more appropriate. In terms of software implementation, string-based assemblers are superior to graph-based ones, of which SOAPdenovo is complex for the creation of configuration file. Our comparison study will assist researchers in selecting a well-suited assembler and offer essential information for the improvement of existing assemblers or the developing of novel assemblers.

## Introduction

In recent years, the next-generation sequencing (or deep sequencing) technologies have been evolving rapidly, with the potential to accelerate biological and biomedical research dramatically [1]. However, the downstream analysis of short reads datasets after sequencing is a tough task; one of the biggest challenges for the analysis of high throughput sequencing reads is the whole genome assembly. DNA fragment assembly has a long history since the emergence of the first generation of sequencing technologies [2], [3]. The assembly procedure becomes especially difficult when tackling short and high throughput reads with different error profiles [4]. According to the existence of reference information, the assembly procedure can be classified as reference-guide genome assembly and *de novo* genome assembly, of which the former is relatively toilless with the aid of reference genome or proteome information while the later in more challenging. Herein, we focus on the comparison and evaluation of tools for *de novo* assembly of genome sequence.

The genome assemblers generally take a file of short sequence reads and a file of quality-value as the input. Since the quality-value file for the high throughput short reads is usually highly memory-intensive, only a few assemblers, for example, SHARCGS [5], and ALLPATHS-LG [6] adopt it in the posterior assembly process. For the sake of computational memory saving and convenience of data inquiry, high-throughput short reads data is always initially formatted to specific data structure. Currently, existing data structure for this usage can be predominantly classified into two categories: string-based model and graph-based model. We therefore call the corresponding assemblers as string-based and graph-based. String-based assemblers, implemented with Greedy-extension algorithm, are mainly reported for the assembly of small genomes [5], [7], [8], [9], while the latter ones are designed aiming at handling complex genomes [10], [11], [12].

One of the most intractable bottlenecks for practical assembly of next - generation short reads is how to process repetitive fragments from complicated genomes, especially eukaryote genomes. Intuitively, sequencing with longer reads is a potential solution, while it becomes impractical with limit current of sequencing technology. The paired-end (PE) sequencing can, to some extent, compensate for read length [13]. Several assemblers, such as SSAKE [9], SOAPdenovo [11], AbySS [12], Velvet [14], [15], exploit PE sequencing information to reduce gaps from assembled contigs. Another big challenge for the assembly of short reads is the intensive computational time requirement. To decrease the time cost of the assembly procedure, thread parallelization is implemented in a couple of graph-based assemblers [11], [12].

**Figure 1:**
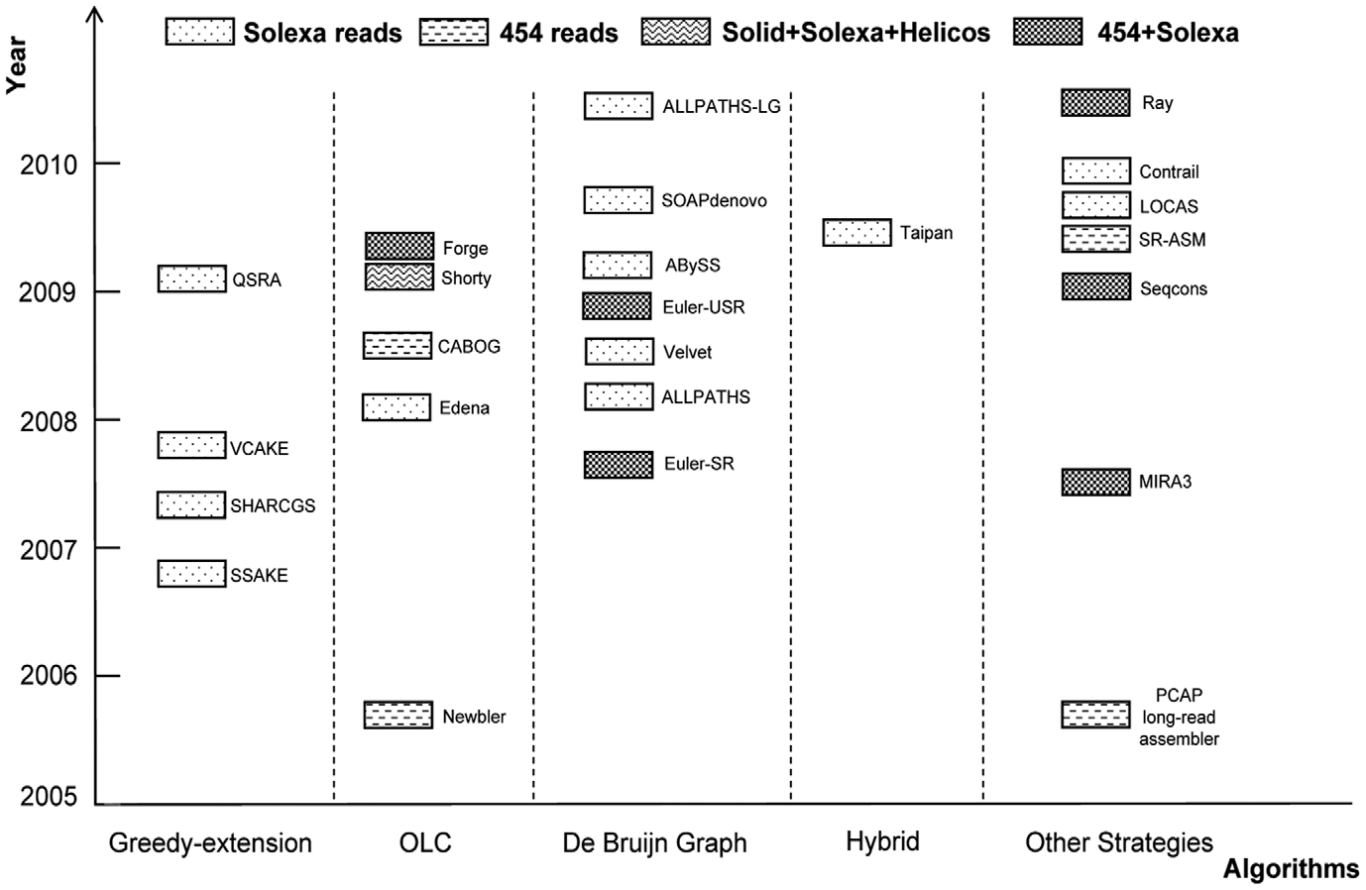
Overview of *de novo* short reads assemblers. Programs developed from year of 2005 to 2010 are classified according to the assembly strategies. Currently, there are mainly four sorts of assemblers, while the other ones are denoted as “Other Strategies”. Different box symbols are utilized to distinguish assemblers that for short reads from different platforms.

At our last enumeration, 24 academic *de novo* genome assemblers, each possessing its own range of application, are developed for short reads datasets from different sequencing platforms in the last few years. These assembly tools with corresponding websites and references are listed in Table S1. We classify and list these assemblers according to their data structure models in Figure 1. In the present study, eight short reads assemblers, representing four various assembly strategies, were benchmarked against two types of simulated short reads datasets derived from four different genomes. Our objective is to gain the assemblers' performance information about computational time and memory cost, assembly accuracy, completeness and size distribution of assembled contigs when each assembler is applied to handle datasets with different data size, then to provide essential information for researchers in choosing suitable tools and for computational biologists to develop novel assemblers. The result indicates that each assembly strategy has its own range of applicability while PE reads and longer reads are indeed with the capability to increase the quality of assembled contigs to some extent, and parallel computing is of great potential in short reads assembly, with which the computational time is notably reduced.

## Results

At present, mainly three distinct strategies are applied in short reads assembly. Among them, Greedy-extension is the implementation of string-based method, while *De Bruijn* graph and overlap-layout-consensus (OLC) are two different graph-based approaches. Each assembly tool is suitable for dataset from specific sequencing platform.

**Figure 2:**
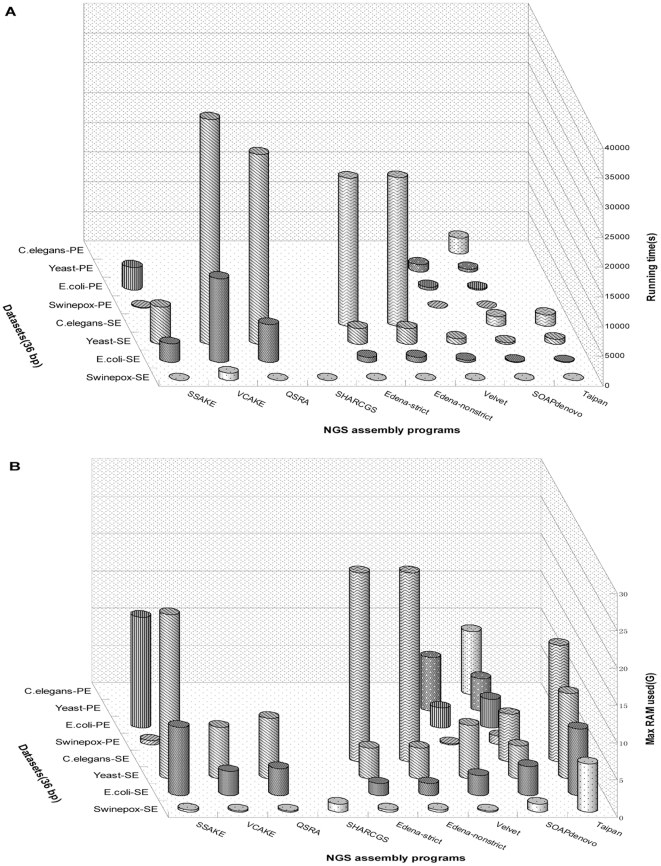
Computational running time and maximum memory occupancy of 36-mer short reads assembly procedures. (A) the computational times of each assembler for different datasets. (B) the maximum RAM used during the assembly process. No data is shown when the RAM is insufficient or the assembly tool is not suitable for the dataset.

**Figure 3:**
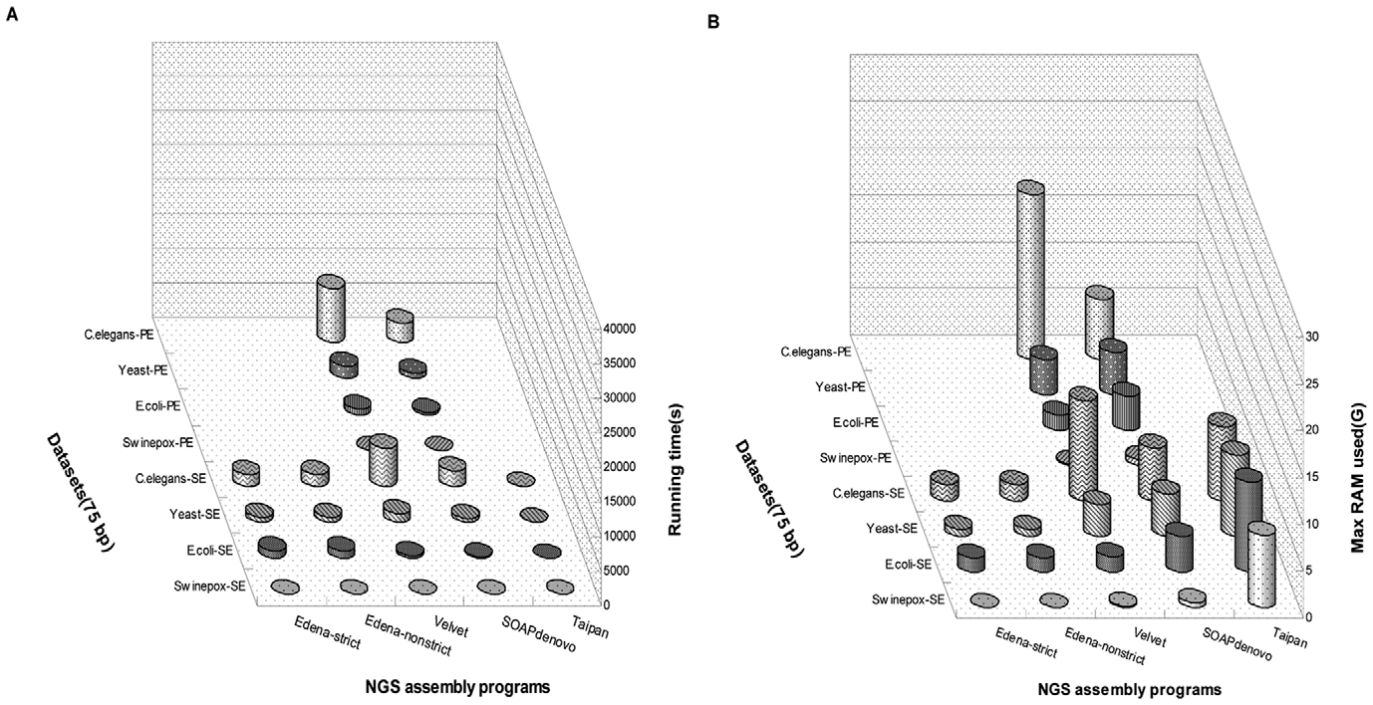
Computational running time and maximum memory occupancy of 75-mer short reads assembly procedures. (A) the computational times of each assembler for different datasets. (B) the maximum RAM used during the assembly process. No data is shown when the RAM is insufficient or the assembly tool is not suitable for the dataset.

For each short reads assembly procedure, less computational time and memory cost is our expectation. The computational time of the assembly process is determined by both the dataset complexity and the assembly strategy. The information about running times, maximum memory occupancies for different assemblers applied to different datasets is illustrated in Figures 2 and 3. For string-based assembler, the time and memory cost is approximately proportionate to dataset size, although it is also affected by the complexity of dataset. Among them, SSAKE runs in rather less time than other peer assemblers, but the RAM usage increases dramatically with augmentation of dataset size. QSRA [7] is developed upon the original VCAKE algorithm, which indeed reduces the computational time, at the cost of RAM occupation. SHARCGS runs in comparable speed as QSRA, however it is highly memory-intensive, even unable to handle *E.coli* short reads dataset with our computer power used in this study. Edena is a typically graph-based assembly tool, which has two running modes: strict and nonstrict modes [16]. For the strict mode, fewer but more accurate contigs are generated, while nonstrict mode acts on the contrary. Compared with string-based tools, Edena is superior in terms of time and RAM utilization. Velvet and SOAPdenovo typify another graph-based method. Similar to Edena, they implement assembly tasks with fairly little computational time and memory usage.

Especially, SOAPdenovo runs in an extreme speed as the exploitation of threads parallelization, but may perform not well enough for small datasets due to the initial task allocation. At last, Taipan was proposed as the hybrid of string-based and graph-based approaches [17], with the dominative feature - the exceedingly short runtime. Nevertheless, the minimum RAM of computer to execute the assembler is high and the requirement for memory grows slowly with the increase of dataset size. Result also shows that more running time and RAM consuming are demanded for paired-end (PE) reads assembly than single-end (SE) reads dataset with the same assembler (Unpublished data). Compare with 36-mer short reads assembly, only OLC, *De Bruijn* and hybrid assemblers can be applied for 75-mer short reads assembly. Our study indicates that no significant difference on the computational time and RAM occupancy for the assembly of these two types of short fragments, with the same sequencing coverage.

**Figure 4:**
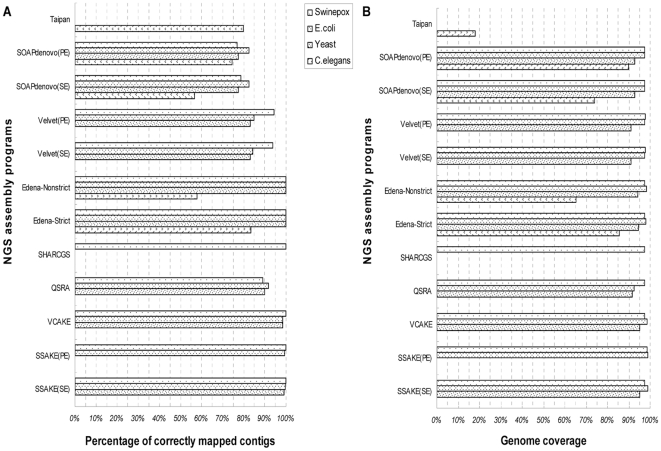
Accuracy and integrity for 36-mer datasets assembly. For short reads assembly, accurate and high genome coverage contigs are expected. Here, the quality of consequential contigs is shown with (A) the accuracy of assembled contigs and (B) the genome coverage of the assembled contigs. No data is shown when the RAM is insufficient or the assembly tool is not suitable for the dataset.

**Figure 5:**
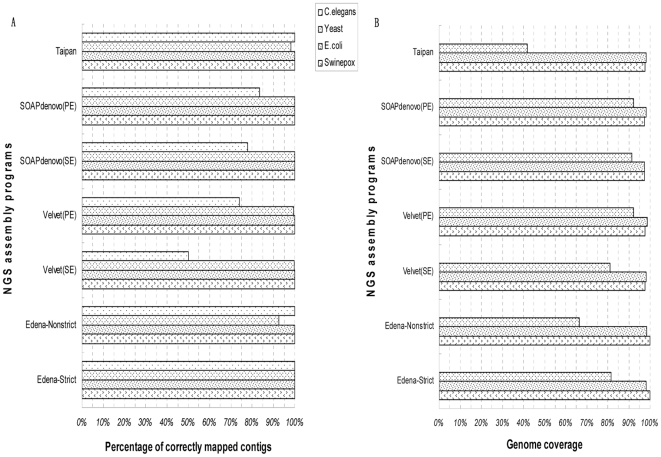
Accuracy and integrity for 75-mer datasets assembly. For short reads assembly, accurate and high genome coverage contigs are expected. Here, the quality of consequential contigs is shown with (A) the accuracy of assembled contigs and (B) the genome coverage of the assembled contigs. No data is shown when the RAM is insufficient or the assembly tool is not suitable for the dataset.

The assembly accuracy and integrity is another consideration for the evaluation of the short reads assemblers. Obviously, contigs with high fidelity and genome coverage are our expectation. Different assemblers have their own performance. Their percentages of correctly mapped contigs and genome coverage for different datasets are shown in Figures 4 and 5. The latest version of SSAKE is of robustness to sequencing errors, compared with it is first version, which was introduced to handle error-free short reads [9]. Other string-based assemblers, such as VCAKE and SHARCGS performed in rivalry with the latest version of SSAKE while QSRA could only generate less precise and lower coverage contigs in contrast with previous tools. What deserves to be mentioned is that Edena, as an assembler based on the overlap-layout-consensus algorithm (OLC) [16], had a quite surpassing performance on various datasets. However, contigs produced from two *De Bruijn* graph-based assemblers, especially SOAPdenovo, were of lower accuracy, but with comparable genome coverage to string-based software. Nevertheless, when handling dataset of huge size, such as short reads from *C.elegans* genome, SOAPdenovo had similar performance as Edena. This result can be elucidated as following: for *De Bruijn* graph-based method, certain proportion of base errors are incorporated into contigs during the construction of graph with k mers generated from input short reads, this process then generate less precise contigs. In the end, the hybrid assembler, Taipan was capable to generate sequences of high accuracy and genome coverage as string-based assembler for small datasets, but performed poorly for the assembly of large genome dataset. After inspection on this assembly procedure, we supposed that it was the exploitation of only partial fraction of short reads that lead to the low coverage productive contigs. Here, we also verified that PE reads is superior to SE reads in terms of resolution for repetitive elements, which is in consistent with previous study [18]. In addition, our result shows that more accurate and higher genome coverage contigs can be produced with longer reads datasets, while it may be a paradox for assembly of large genome, such as *C.elegans*, of which none of the selected assemblers in this study is suitable for its 75-mer reads assembly.

**Figure 6:**
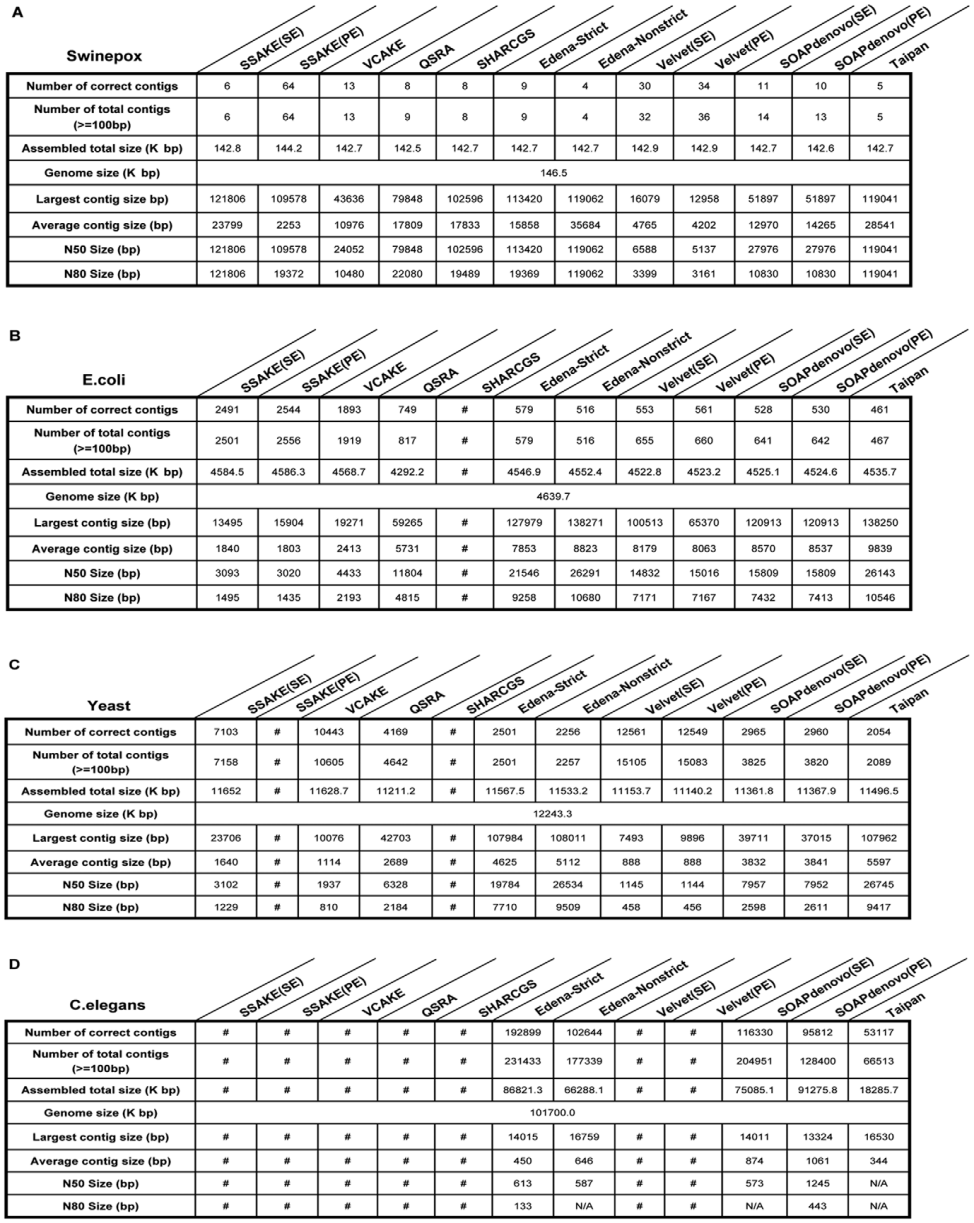
Statistics for assembled contigs of 36-mer short reads. Indicatrix that illustrates the feature of size distribution are adopted for analysis. “#” denotes the RAM of machine is not enough, and “N/A” means the data is not available. The N50 size and N80 size represent the maximum read length for which all contigs greater than or equal to the threshold covered 50% or 80% of the reference genome.

**Figure 7:**
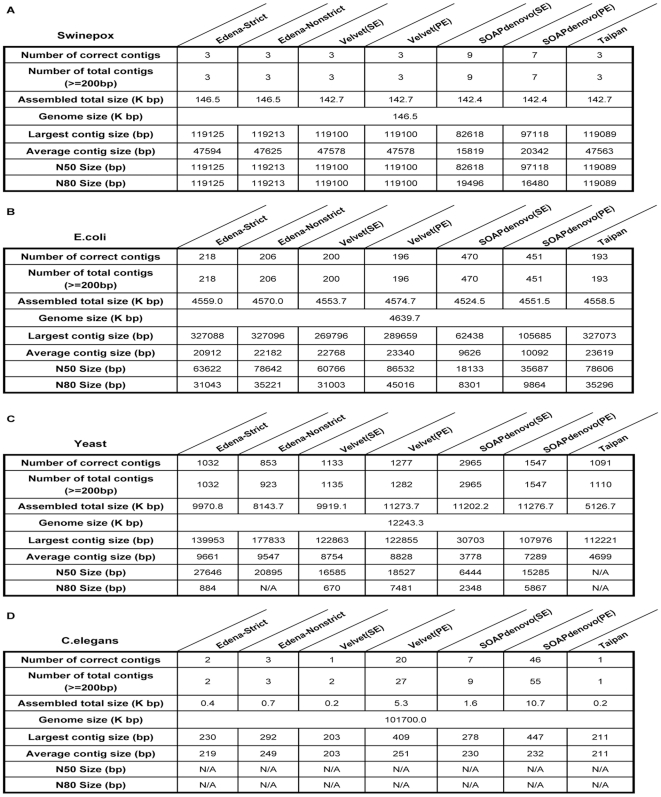
Statistics for assembled contigs of 75-mer short reads. Indicatrix that illustrates the feature of size distribution are adopted for analysis. “#” denotes the RAM of machine is not enough, and “N/A” means the data is not available. The N50 size and N80 size represent the maximum read length for which all contigs greater than or equal to the threshold covered 50% or 80% of the reference genome.

For further analysis of assembled contigs, the contig size distribution was calculated and shown in Figures 6 and 7. For many biological studies, DNA sequence with sufficient length is necessary. Under ideal condition, only one contig that matches the whole genome sequence perfectly could be generated from each assembly procedure. Practically, the contigs generated by different assembly procedures are separated by gaps for the presence of repetitive fragments. From Figures 2, 3, 4, 5, 6, and 7, it is clear that different assembly strategies perform differently on diverse datasets. For dataset of very small size, string-based assemblers produced fewer but longer reads than *De Bruijn* graph-based tools. However, it became reverse when the size of dataset increases. Edena, the OLC assembler, could assemble short reads into relatively long contigs for various datasets. Taipan, as a hybrid assembly tool, had better performance than Edena for small datasets. When handling short fragments from large genomes such as *C.elegans*, even though fairly longer largest contigs was formed, N50 and N80 size were not available with too few assembled contigs. Here in general, we can claim that PE reads or longer reads would generate better assembly results. Besides, for *De Bruijn* assemblers, Velvet produced better assembly result than SOAPdenovo when assembly of 75-mer short reads datasets, because of the wider range of K value to be chosen in Velvet.

## Discussion

Even though the assembly algorithms for *de novo* genome assembly have been well-reviewed [19], we are the first to test and compare these tools with different datasets practically. The key concern for the assessment of an assembler is its usability and assembly quality. We evaluated the current assemblers from the two aspects with simulated Solexa short reads datasets (the detail could be found in the method section) on one single server machine, as Solexa/IIIumina sequencing technology is the most widely applied technology. Our results show that string-based assemblers, OLC assemblers are well-suited for very short reads and longer reads respectively for small genome comprising millions of short reads, when the computational power is limited. But Taipan is a better choice for its excellent assembly speed if the RAM of machine is sufficient. For large datasets of more than hundred millions of short reads, *De Bruijn* graph-based assemblers could have commendable resolutions due to their short runtime and low RAM occupancy, of which SOAPdenovo performs well on very short reads, while ALLPATHS-LG could be a good choice for ∼100 bp short reads assembly, as it was described [6]. In terms of ease of software installation, string-based assemblers and hybrid assembler are superior to graph-based ones, of which SOAPdenovo is complex for the creation of configuration file. In addition, as shown in this study, new assemblers for longer reads are much-needed, since the majority ones were designed for very short reads. Recommended assemblers for different assembly processes are shown in Table 1.

**Table 1:** Recommended assemblers for different genome assembly^1^.

	Type of reads	RAM of Machine	Recommended assembler
**Small genome**(Microorganism)	Very short(36 bp)	Large (>16G)	Hybrid assembler: Taipan
		Small (<16G)	SSAKE, QSRA, Edena
	Short(75 bp)	Large (>16G)	Hybrid assembler: Taipan
		Small (<16G)	OLC assembler: Edena
**Large genome**(Eukaryote)	Very short(36 bp)	Large (>16G)	De Bruijn assembler: SOAPdenovo
		Small (<16G)	—
	Short(75 bp)	Large (>16G)	De Bruijn assembler: ALLPATHS-LG
		Small (<16G)	—

1 According to our evaluation study, the specific assembler is recommended for different type of assembly procedure. Herein, only tools running on a single machine are considered, while other assemblers running on cluster machines, such as ABySS and Ray, may also perform well for large genome assembly.

Assembly for small genomes, such as prokaryote organisms, has been well resolved [20], [21], [22]. However, short reads from eukaryote genomes, with features of gargantuan size and high repetitiveness, make sky-high requirement for assembly strategies and computer hardware [10], [11], [23], [24]. Exceptional data storage methods are required to reduce RAM occupancy, for example, ABySS transfers the sequence reads into binary format to save the computational space [12]. Threads parallelization is a solution to accelerate assembly speed. Three hierarchies of parallelization are taken into implementation: multi-thread on a single machine [11], multi-process with cluster machines [12], [25] and cloud computation (http://sourceforge.net/apps/mediawiki/contrail-bio/index.php?title=Contrail). Interestingly, GPU computational method has been applied in other two short reads analysis procedures, *i.e.* error correction and alignment, and speeded up of these processes many times as reported [26], [27], [28]. Great improvement may be expected with the application of this approach in assembly process afterwards. Besides, integration of multi datasets from various sequencing strategies are exploited to tackle the complex genome assembly [29], which greatly challenge the development of assembly algorithms to suit for diverse short reads. Usually, several assembler are combined for this issue [30]. Meanwhile, the accuracy and read length of sequenced tags are increasing stepwisely, and PE sequencing strategies are extensively carried out on different next generation sequencing (NGS) platforms. With the cooperation between biologist, bioinformaticians and developers of high performance machine, we can expect that *de novo* assembly of short reads will be less challenging for NGS data analysis in the near future.

## Methods

### Short reads data simulation

**Figure 8:**
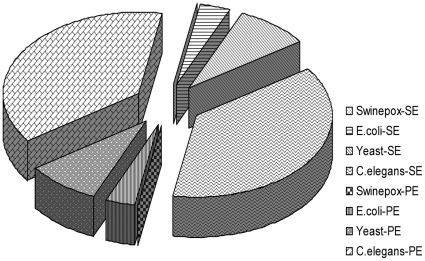
Size comparison of datasets used in this study. This figure shows the relative size comparison of short reads datasets with different legends. SE denotes Single-end short reads dataset, while PE denotes Paired-end short reads dataset.

**Figure 9:**
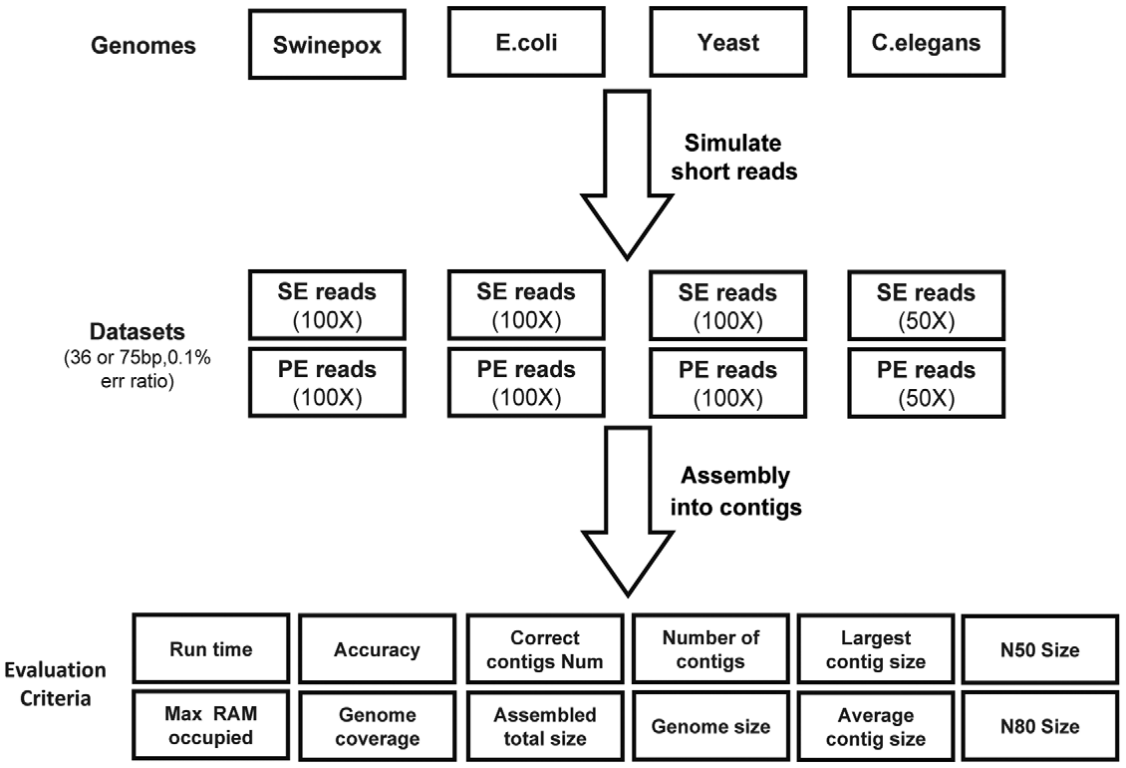
Pipeline for evaluation of short reads assembly programs. Four reference genomes with different size are exploited to generate short reads bearing base errors. The performance of assemblers is evaluated through computational time, accuracy, integrity and contig size, etc.

To get the precise information about the quality of assembled results, we simulated the short reads datasets sequencing from Solexa/IIIumina with the perl script program (see Package S1), for the reason that there is no exact genome sequence for real sequenced datasets. Currently, the real data from Solexa platform is 75 bp per read, while the 36 bp sequencing mode is still well-supported. According to the report by Jay Shendure & Hanlee [1], the dominant error type for Solexa sequencing protocol is substitution, and the error rate of 0.1% could be achieved after quality filtering. Hence, in this work, we only consider substitution error type and adopt 0.1% error rate, even though which may change slightly as sequencing technology develops. The Swinepox virus (Swinepox), *Escherichia coli* str. K-12 substr (*E.coli*), *Saccharomyces cerevisiae* (Yeast) and *Caenorhabditis elegans* (*C.elegans*) genomes were downloaded from Genebank (Genebank accession number NC_003389, NC_000913, NC_001133–NC_001148, NC_003279–NC_003284) respectively. SE reads dataset and PE reads dataset with length of 36 bp and 75 bp were simulated according to each genome sequence. For SE reads, all possible 36mers (or 75 mers) were extracted from both strands for these genomes then added an error rate of 0.1% to the generated reads. Sequences were selected at random to simulate up to 100× read coverage for the first three genomes and up to 50× coverage for *C.elegans* genome. For PE reads, simulated sequences were generated by sliding window approach with an (R+2r) bp window size and 1 bp step size (R is 2000 for *C.elegans*, 500 for 3 other genomes, r is the short read size). Along each genome reference sequence the first 36 bp (or 75 bp) and the reverse complement of the last 36 bp (or 75 bp) in each window frame were collected than add an error rate of 0.1% to the reads. PE read datasets with the same read coverage as from SE reads synthesis procedure were generated. The size comparison of these datasets is shown in Figure 8; Figure 9 displays the pipeline of the whole evaluation study.

### Preliminary analysis of reference genome sequence

**Figure 10:**
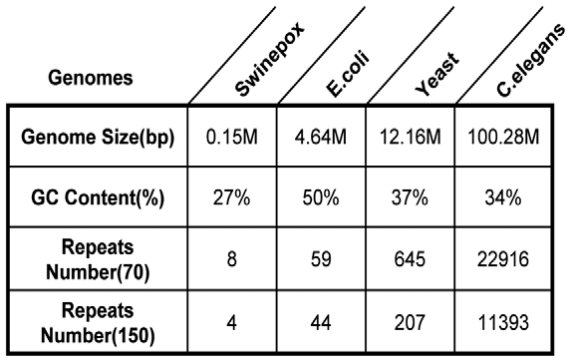
Analysis of complexity of reference genome sequences. Tandem repeats finder (Version 4.04) is utilized to detect the number of repeat elements with length less than 2000 bp, the parameter “minimum alignment score ” is set to 70 and 150 for two types of short reads. The increase of genome size, repeat numbers and GC content may imply the increasing in genome assembly complexity.

Genome sequence assembly is greatly challenged by repeat sequences, especially when the repeats are longer than short reads [4]. To address this issue, longer reads and PE information are required [13]. Before the assembly procedure implemented, we detected the repeat elements in reference genomes with Tandem Repeats Finder [31]. The number of repeats reflects the complexity of target sequence to a large extent (see Figure 10).

### Program implementation

**Figure 11:**
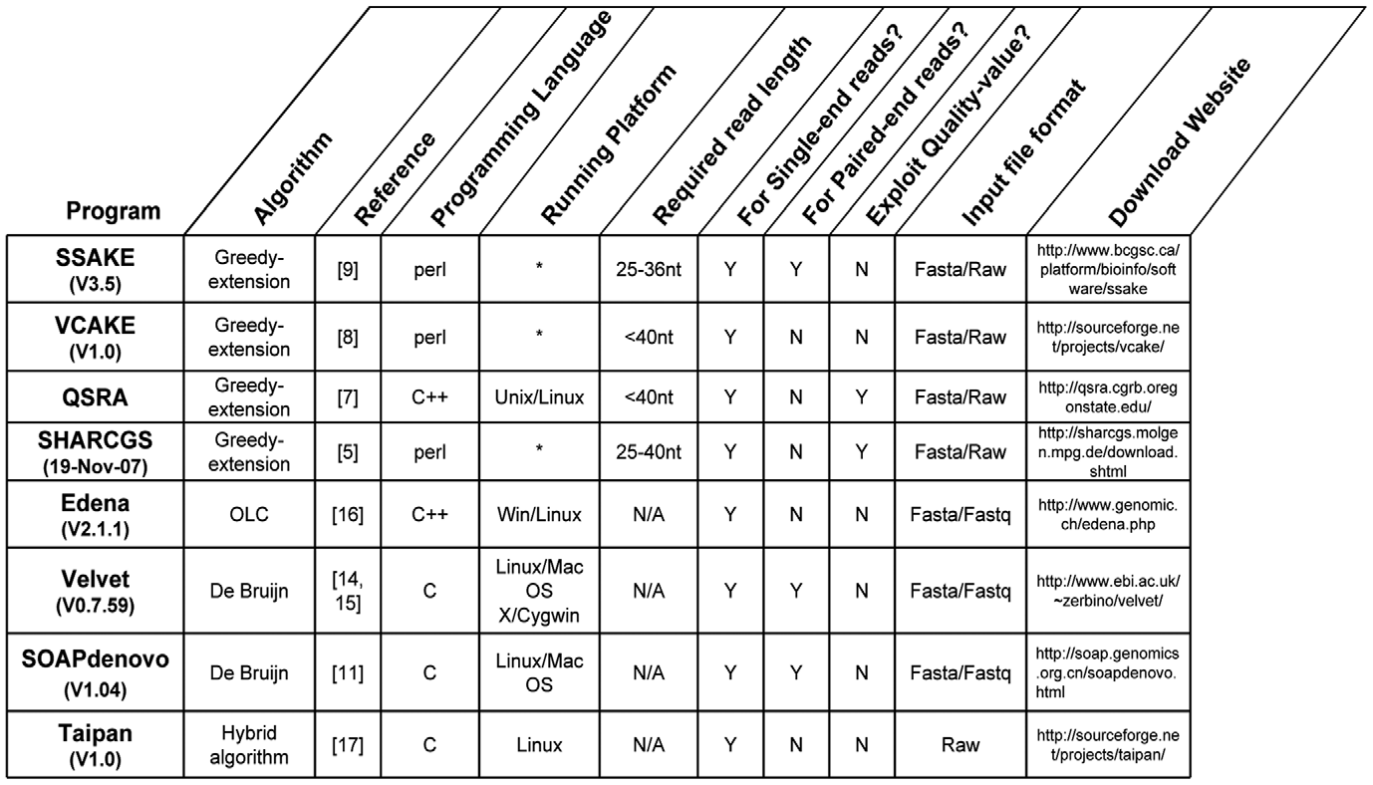
Features of selected short reads assembly programs. Noncommercial programs based on varied sorts of assembly approaches were selected for testing on synthetic Solexa short reads. “*” indicates any operating systems with perl interpreter, while “OLC” is for overlap-layout-consensus, and “N/A” for not available. The features for different programs are obtained from related references and documents of the latest version software (version information is not listed here).

Eight short reads assembly programs (see Figure 11), which represent 4 different assembly strategies, were selected for assembly of simulated short reads. For each assembly procedure, we set 3 different series of parameters (Table S2), from which the best assembly result was chosen for the evaluation of the performance of each program respectively.

All the selected programs were run on a server machine equipped with four 2.4GHz Intel(R) Xeon(R) 4 CPU, 4 cores within each CPU, and 32 GB of RAM. The operating system is Ubuntu 8.04.4 with version of X_86 64 bit.

### Performance evaluation

The computational time consuming and maximum memory occupancy during each assembly procedure are recorded with a perl and python script. The mean of the computational time and computational memory cost of three processes with different parameters was considered to be the performance of each corresponding assembler. Data was not shown when the machine memory is insufficient or the assembler is not suitable for the dataset.

### Accuracy and integrity

Contigs generated from each assembly process were mapped to each homologous genome reference sequence with NCBI Blast-2.2.20 for Windows 32bit machine [32], of which with size no shorter than Ybp (Y is 100 bp for 36mer datasets and 200 bp for 75mer datasets) and at least 98% of each read completely match to the reference sequence were presumed to be correct. We calculate the accuracy with *Acc* = NC/N, where NC and N represent the number of correct contigs and the number of contigs longer than Ybp respectively. The integrity was computed with equation *Inte* = (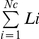)/L, which means the ratio of the sum of all the correct contig sizes to the reference sequence size. Outcome with optimal accuracy and integrity was chosen as the best performance of each assembler.

### Statistical information of assembled contigs

To further evaluate the performance of each assembly tool, we also provide the information of size distribution of assembled contigs, including number of correct contigs, number of total assembled contigs, largest contig size, average contig size, N50 and N80 contig sizes. The N50 and N80 represent the size N such that 50% or 80% of the genome is contained in contigs of size N or greater. With this information, we can compare and measure the genome assemblies statistically.

## Supporting Information

Table S1 **The websites and references for de** ***novo*** **NGS assemblers.**
(DOC)

Table S2 **The parameters for each assembly procedure.** For each pair of assembler and dataset, 3 groups of parameter are adopted for short reads assembly. Symbol “—” and “*Out of RAM*” means the assembler does not suit for corresponding type of dataset and memory required for the assembly process is beyond computer power, while parameters in bold will be the best for the assembly.
(DOC)

Package S1 **The perl scripts and the test file used to simulate the short reads .** We modified the program written by Juliane Dohm and Claudio Lottaz to simulate both single-end reads and paired-end reads from a given reference sequence.
(RAR)
