# Supplementary material for: A Practical Comparison of De Novo Genome Assembly Software Tools for Next-Generation Sequencing Technologies
Source: PLoS One. 2011 Mar 14;6(3):e17915. doi: 10.1371/journal.pone.0017915 (PMC3056720; doi:10.1371/journal.pone.0017915)
Supplement: Table S1 — The websites and references for de novo NGS assemblers. (DOC) [file pone.0017915.s001.doc]

**Table S1 Detailed information for de novo NGS assemblers**

| **Program** | **Reference** | **Website** |
| --- | --- | --- |
| SSAKE | [1] | http://www.bcgsc.ca/bioinfo/software/ssake |
| VCAKE | [2] | http://sourceforge.net/projects/vcake/ |
| QSRA | [3] | http://qsra.cgrb.oregonstate.edu |
| SHARCGS | [4] | http://sharcgs.molgen.mpg.de |
| Newbler | [5] | http://454.com/contact-us/software-request.asp |
| Edena | [6] | http://www.genomic.ch/edena |
| CABOG | [7] | http://wgs-assemblers.sf.net |
| Shorty | [8] | http://www.cs.sunysb.edu/~skiena/shorty |
| Euler-SR | [9] | http://euler-assembler.ucsd.edu/portal/ |
| ALLPATHS | [10, 11] | ftp://ftp.broad.mit.edu/pub/crd/ALLPATHS/Release-3-0 |
| ALLPATHS-LG | [12] | ftp://ftp.broadinstitute.org/pub/crd/ALLPATHS/Release-LG/ |
| Velvet | [13, 14] | http://www.ebi.ac.uk/~zerbino/velvet |
| Euler-USR | [15] | http://euler-assembler.ucsd.edu/portal/ |
| ABySS | [16, 17] | http://www.bcgsc.ca/downloads/abyss/abyss-1.1.2.tar.gz |
| SOAPdenovo | [18] | http://soap.genomics.org.cn/soapdenovo.html |
| Taipan | [19] | http://taipan.sourceforge.net |
| PCAP long-read assembler | [20] | http://seq.cs.iastate.edu/pcap.html |
| MIRA3 | N/A | http://sourceforge.net/projects/mira-assembler/files/ |
| Seqcons | [21] | http://www.seqan.de/uploads/media/MicroRazerS.zip |
| Forge | [22] | http://sourceforge.net/projects/forge |
| SR-ASM | N/A | https://ngslib.genome.tugraz.at/node/13 |
| LOCAS | N/A | www-ab.informatik.uni-tuebingen.de/software/locas |
| Contrail | N/A | http://sourceforge.net/apps/mediawiki/contrail-bio/index.php?title=Contrail |
| Ray | [23] | http://sourceforge.net/projects/denovoassembler/files/ |

**References**

1. Warren RL, Sutton GG, Jones SJ, Holt RA (2007) Assembling millions of short DNA sequences using SSAKE. Bioinformatics 23(4): 500-501.

2. Jeck WR, Reinhardt JA, Baltrus DA, Hickenbotham MT, Magrini V, et al. (2007) Extending assembly of short DNA sequences to handle error. Bioinformatics 23(21): 2942-2944.

3. Bryant DW Jr, Wong WK, Mockler TC (2009) QSRA: a quality-value guided de novo short read assembler. BMC Bioinformatics 10(1): 69-75.

4. Dohm JC, Lottaz C, Borodina T, Himmelbauer H (2007) SHARCGS, a fast and highly accurate short-read assembly algorithm for de novo genomic sequencing. Genome Res 17(11): 1697-1706.

5. Margulies M, Egholm M, Altman WE, Attiya S, Bader JS, et al. (2005) Genome sequencing in microfabricated high-density picolitre reactors. Nature 437(7057): 376-380.

6. Hernandez D, Francois P, Farinelli L, Osteras M, Schrenze J (2008) De novo bacterial genome sequencing: millions of very short reads assembled on a desktop computer. Genome Res 18(5): 802-809.

7. Miller JR, Delcher AL, Koren S, Venter E, Walenz BP, et al. (2008) Aggressive assembly of pyrosequencing reads with mates. Bioinformatics 24(24): 2818-2824.

8. Hossain MS, Azimi N, Skiena S (2009) Crystallizing short-read assemblies around seeds. BMC Bioinformatics 10 (Suppl 1): S1-S16.

9. Chaisson MJ and Pevzner PA (2008) Short read fragment assembly of bacterial genomes. Genome Res 18(2): 324-330.

10. Butler J, MacCallum I, Kleber M, Shlyakhter IA, Belmonte MK, et al. (2008) ALLPATHS: de novo assembly of whole-genome shotgun microreads. Genome Res 18(5): 810-820.

11. Maccallum I, Przybylski D, Gnerre S, Burton J, Shlyakhter I, et al. (2009) ALLPATHS 2: small genomes assembled accurately and with high continuity from short paired reads. Genome Biol 10(10): R103.

12. Gnerre S, Maccallum I, Przybylski D, Ribeiro FJ, Burton JN, et al. (2010) High-quality draft assemblies of mammalian genomes from massively parallel sequence data. PNAS: 10.1073

13. Zerbino DR, Birney E (2008) Velvet: algorithms for de novo short read assembly using de Bruijn graphs. Genome Res 18(5): 821-829.

14. Zerbino DR, McEwen GK, Margulies EH, Birney E (2009) Pebble and rock band: heuristic resolution of repeats and scaffolding in the velvet short-read de novo assembler. PLoS One 4(12): e8407.

15. Chaisson MJ, Brinza D, Pevzner PA (2009) De novo fragment assembly with short mate-paired reads: Does the read length matter? Genome Res 19(2): 336-346.

16. Simpson JT, Wong K, Jackman SD, Schein JE, Jones SJ, et al. (2009) ABySS: a parallel assembler for short read sequence data. Genome Res 19(6): 1117-1123.

17. Birol I, Jackman SD, Nielsen CB, Qian JQ, Varhol R, et al. (2009) De novo transcriptome assembly with ABySS. Bioinformatics 25(21): 2872-2877.

18. Li R, Zhu H, Ruan J, Qian W, Fang X, et al. (2009) De novo assembly of human genomes with massively parallel short read sequencing. Genome Res 20(2): 265-272.

19. Schmidt B, Sinha R, Beresford SB, Puglisi SJ (2009) A fast hybrid short read fragment assembly algorithm. Bioinformatics 25(17): 2279-2280.

20. Huang X, Yang SP (2005) Generating a genome assembly with PCAP. Curr Protoc Bioinformatics, Chapter 11: p. Unit11 3.

21. Rausch T, Koren S, Denisov G, Weese D, Emde AK, et al. (2009) A consistency-based consensus algorithm for de novo and reference-guided sequence assembly of short reads. Bioinformatics 25(9): 1118-1124.

22. Diguistini S, Liao NY, Platt D, Robertson G, Seidel M, et al. (2009) De novo genome sequence assembly of a filamentous fungus using Sanger, 454 and Illumina sequence data. Genome Biol 10(9): R94.

23. Boisvert S, Laviolette F, Corbeil J (2010) Ray: simultaneous assembly of reads from a mix of high-throughput sequencing technologies. J Comput Biol 17(11): 1519-1533.
