# Supplementary material for: A Practical Comparison of De Novo Genome Assembly Software Tools for Next-Generation Sequencing Technologies
Source: PLoS One. 2011 Mar 14;6(3):e17915. doi: 10.1371/journal.pone.0017915 (PMC3056720; doi:10.1371/journal.pone.0017915)
Supplement: Table S2 — The parameters for each assembly procedure. For each pair of assembler and dataset, 3 groups of parameter are adopted for short reads assembly. Symbol “—” and “Out of RAM” means the assembler does not suit for corresponding type of dataset and memory required for the assembly process is beyond computer power, while parameters in bold will be the best for the assembly. (DOC) [file pone.0017915.s002.doc]

| **Table S2. The parameters for each assembly procedure** | | | | | | | | | | |
| --- | --- | --- | --- | --- | --- | --- | --- | --- | --- | --- |
| For each combination of assembler and dataset, 3 groups of parameter are adopted for short reads assembly.  Symbol “---” and “*Out of RAM*” means the assembler does not suit for corresponding type of dataset and memory required for the  assembly process is beyond machine power, while parameters in bold style produce the best assembly result. | | | | | | | | | | |
| **Dataset** | | **SSAKE**  **(V3.5)** | **VCAKE**  **(V1.0)** | **QSRA** | **SHARCGS**  **(19-Nov-07)** | **Edena**  **-strict**  **(V2.1.1)** | **Edena**  **-nonstrict**  **(V2.1.1)** | **Velvet**  **(V0.7.59)** | **SOAPdenovo**  **(V1.04)** | **Taipan**  **(V1.0)** |
| Swinepox | SE  reads  (36bp) | **m=20** | e=20 | **u=20** | **r=2** | M=20 | M=20 | **K=19** | **K=21 p=8** | **K=20** |
|  |  | m=25 | **e=25** | u=25 | r=3 | **M=25** | M=25 | K=25 | K=25 p=8 | K=25 |
|  |  | m=30 | e=30 | u=30 | r=4 | M=30 | **M=30** | K=29 | K=31 p=8 | K=30 |
|  | PE  reads  (36bp) | m=20 d=500 | --- | --- | --- | --- | --- | **K=19 ins_len=500** | **K=21 p=8** | --- |
|  |  | **m=25 d=500** | --- | --- | --- | --- | --- | K=25 ins_len=500 | K=25 p=8 | --- |
|  |  | m=30 d=500 | --- | --- | --- | --- | --- | K=29 ins_len=500 | K=31 p=8 | --- |
|  | SE  reads  (75bp) | --- | --- | --- | --- | M=40 | M=40 | K=39 | K=21 p=8 | K=40 |
|  |  | --- | --- | --- | --- | **M=55** | **M=55** | **K=55** | K=25 p=8 | **K=55** |
|  |  | --- | --- | --- | --- | M=70 | M=70 | K=69 | **K=31 p=8** | K=70 |
|  | PE  reads  (75bp) | --- | --- | --- | --- | --- | --- | K=39 ins_len=500 | K=21 p=8 | --- |
|  |  | --- | --- | --- | --- | --- | --- | **K=55 ins_len=500** | K=25 p=8 | --- |
|  |  | --- | --- | --- | --- | --- | --- | K=69 ins_len=500 | **K=31 p=8** | --- |
| E.coli | SE  reads  (36bp) | m=20 | e=20 | u=20 | *Out of RAM* | M=20 | M=20 | K=19 | K=21 p=8 | K=20 |
|  |  | **m=25** | e=25 | u=25 |  | M=25 | M=25 | **K=25** | **K=25 p=8** | **K=25** |
|  |  | m=30 | **e=30** | **u=30** |  | **M=30** | **M=30** | K=29 | K=31 p=8 | K=30 |
|  | PE  reads  (36bp) | m=20 d=500 | --- | --- | --- | --- | --- | K=19 ins_len=500 | K=21 p=8 | --- |
|  |  | **m=25 d=500** | --- | --- | --- | --- | --- | **K=25 ins_len=500** | **K=25 p=8** | --- |
|  |  | m=30 d=500 | --- | --- | --- | --- | --- | K=29 ins_len=500 | K=31 p=8 | --- |
|  | SE  reads  (75bp) | --- | --- | --- | --- | M=40 | M=40 | K=39 | K=21 p=8 | **K=40** |
|  |  | --- | --- | --- | --- | **M=55** | **M=55** | **K=55** | K=25 p=8 | K=55 |
|  |  | --- | --- | --- | --- | M=70 | M=70 | K=69 | **K=31 p=8** | K=70 |
|  | PE  reads  (75bp) | --- | --- | --- | --- | --- | --- | K=39 ins_len=500 | K=21 p=8 | --- |
|  |  | --- | --- | --- | --- | --- | --- | **K=55 ins_len=500** | K=25 p=8 | --- |
|  |  | --- | --- | --- | --- | --- | --- | K=69 ins_len=500 | **K=31 p=8** | --- |
| Yeast | SE  reads  (36bp) | m=20 | e=20 | u=20 | *Out of RAM* | M=20 | M=20 | **K=19** | **K=21 p=8** | K=20 |
|  |  | **m=25** | **e=25** | u=25 |  | M=25 | M=25 | K=25 | K=25 p=8 | **K=25** |
|  |  | m=30 | e=30 | **u=30** |  | **M=30** | **M=30** | K=29 | K=31 p=8 | K=30 |
|  | PE  reads  (36bp) | *Out of RAM* | --- | --- | --- | --- | --- | **K=19 ins_len=500** | **K=21 p=8** | --- |
|  |  |  | --- | --- | --- | --- | --- | K=25 ins_len=500 | K=25 p=8 | --- |
|  |  |  | --- | --- | --- | --- | --- | K=29 ins_len=500 | K=31 p=8 | --- |
|  | SE  reads  (75bp) | --- | --- | --- | --- | **M=40** | **M=40** | **K=39** | K=21 p=8 | **K=40** |
|  |  | --- | --- | --- | --- | M=55 | M=55 | K=55 | **K=25 p=8** | K=55 |
|  |  | --- | --- | --- | --- | M=70 | M=70 | K=69 | K=31 p=8 | K=70 |
|  | PE  reads  (75bp) | --- | --- | --- | --- | --- | --- | **K=39 ins_len=500** | K=21 p=8 | --- |
|  |  | --- | --- | --- | --- | --- | --- | K=55 ins_len=500 | K=25 p=8 | --- |
|  |  | --- | --- | --- | --- | --- | --- | K=69 ins_len=500 | **K=31 p=8** | --- |
| C.elegans | SE  reads  (36bp) | *Out of RAM* | *Out of RAM* | *Out of RAM* | *Out of RAM* | **M=20** | M=20 | *Out of RAM* | **K=21 p=8** | K=20 |
|  |  |  |  |  |  | M=25 | M=25 |  | K=25 p=8 | **K=25** |
|  |  |  |  |  |  | M=30 | **M=30** |  | K=31 p=8 | K=30 |
|  | PE  reads  (36bp) | *Out of RAM* | --- | --- | --- | --- | --- | *Out of RAM* | K=21 p=8 | --- |
|  |  |  | --- | --- | --- | --- | --- |  | K=25 p=8 | --- |
|  |  |  | --- | --- | --- | --- | --- |  | **K=31 p=8** | --- |
|  | SE  reads  (75bp) | --- | --- | --- | --- | M=40 | **M=40** | **K=39** | K=21 p=8 | **K=40** |
|  |  | --- | --- | --- | --- | **M=55** | M=55 | K=55 | K=25 p=8 | K=55 |
|  |  | --- | --- | --- | --- | M=70 | M=70 | K=69 | **K=31 p=8** | K=70 |
|  | PE  reads  (75bp) | --- | --- | --- | --- | --- | --- | **K=39 ins_len=2000** | K=21 p=8 | --- |
|  |  | --- | --- | --- | --- | --- | --- | K=55 ins_len=2000 | K=25 p=8 | --- |
|  |  | --- | --- | --- | --- | --- | --- | K=69 ins_len=2000 | **K=31 p=8** | --- |

**Definition for parameters:** For SSAKE, m denotes minimum overlapping length for contig extension, while the value of d is for the insert size of paired-end reads. For VCAKE, QSRA, Edena and Taipan, parameters - e, u, M and K also indicate the minimun overlapping size for extension, just as m in SSAKE. Parameter r in SHARCGS denotes minimum read repetition for quality filtering of short reads. And K value of Velvet and SOAPdenovo signify K-mer size for *De Bruijn* graph construction, while ins_len and p denote insert size for paired-end reads and number of threads for parallel computing, respectively. According to document of Velvet, E(x) = C*(L-K+1)/L, where C, K, L denotes sequencing coverage, K-mer size and read length, the value of E(x) is generally between 10 and 20. Herein, as the MAXKMERSIZE of Velvet can be adjusted, K values for which are chosen for different size of short reads .For SOAPdenovo, the same K value is used, since range of K value is fixed in the pre-compiled program.
